# Risk factors for synchronous high-risk polyps in patients with colorectal cancer

**DOI:** 10.3389/fsurg.2024.1424809

**Published:** 2024-06-24

**Authors:** Degao He, Junguo Chen, Xuefei Jiang, Hao Chen, Juanni Huang, Zexian Chen

**Affiliations:** ^1^ Department of General Surgery (Colorectal Surgery), The Sixth Affiliated Hospital, Sun Yat-sen University, Guangzhou, Guangdong, China; ^2^ Guangdong Provincial Key Laboratory of Colorectal and Pelvic Floor Diseases, The Sixth Affiliated Hospital, Sun Yat-sen University, Guangzhou, Guangdong, China; ^3^ Biomedical Innovation Center, The Sixth Affiliated Hospital, Sun Yat-sen University, Guangzhou, Guangdong, China; ^4^ Department of Anorectal Surgery, Shenzhen Longhua District Central Hospital, Shenzhen, Guangdong, China; ^5^ Department of Geriatrics, The First Affiliated Hospital of Guangzhou Medical University, Guangzhou, Guangdong, China

**Keywords:** high-risk polyps, colorectal cancer, risk factors, postoperative surveillance, colonoscopy

## Abstract

**Purpose:**

Colorectal cancer (CRC) patients may experience inadequate preoperative colonoscopy due to bowel obstruction or inadequate bowel preparation, leading to potential oversight of other polyps. We aimed to identify risk factors for CRC complicated with synchronous high-risk polyps.

**Methods:**

A retrospective analysis of 6,674 CRC patients from December 2014 to September 2018 was conducted. High-risk polyps were defined as adenomas or serrated polyps that were ≥10 mm, or with tubulovillous/villous components or high-grade dysplasia. All other polyps were defined as low-risk polyps. Patients with complete pathological and clinical information were categorized into three groups: the no polyp group, the low-risk polyp group, and the high-risk polyp group. Univariate and multivariate logistic regression analyses were performed to calculate the odds ratios (ORs) and corresponding 95% confidence intervals (CIs) for all potential risk factors.

**Results:**

Among the 4,659 eligible patients, 848 (18.2%) were found to have low-risk polyps, while 675 (14.5%) were diagnosed with high-risk polyps. In a multivariate logistic regression model, compared to patients without polyps, those with synchronous high-risk polyps were more likely to be male (OR = 2.07), aged 50 or older (OR = 2.77), have early-stage tumors (OR = 1.46), colon tumors (OR = 1.53), NRAS mutant tumors (OR = 1.66), and BRAF wild-type tumors (OR = 2.43).

**Conclusion:**

Our study has identified several risk factors associated with the presence of synchronous high-risk polyps in CRC patients. Based on these findings, we recommend that patients who exhibit these high-risk factors undergo early follow-up of colonoscopy to detect synchronous polyps early.

## Introduction

Colorectal cancer (CRC) is the third most common cancer and the second leading cause of cancer-related death worldwide (1). The majority of CRC cases originate from premalignant polyps that accumulate sufficient mutations to progress towards high-grade dysplasia and subsequently CRC (2). Premalignant polyps encompass both conventional adenomas and serrated polyps. Conventional adenomas are further classified into tubular adenomas, tubulovillous adenomas, and villous adenomas, while serrated polyps are classified as traditional serrated adenomas, sessile serrated polyps, and hyperplastic polyps according to the WHO classification (3, 4). The removal of premalignant polyps represents the most effective approach for CRC prevention and may hold greater significance compared to subsequent surveillance measures (5).

The quality of colonoscopy significantly influences the detection of colorectal lesions. European Society of Gastrointestinal Endoscopy (ESGE) and European Society of Digestive Oncology (ESDO) Guidelines recommend high-quality perioperative colonoscopy to identify and remove synchronous lesions before CRC surgery (6, 7). However, in cases where CRC patients experience bowel obstruction, the endoscope may be unable to pass through the tumor to visualize the proximal bowel, potentially leading to missed lesions such as synchronous polyps. Meanwhile, poor bowel preparation can also lead to incomplete colonoscopy. Incomplete preoperative colonoscopy is associated with an elevated risk of postoperative CRC, particularly metachronous CRC (mCRC). mCRC is defined as a second primary CRC diagnosed at least 6 months after the initial CRC diagnosis, rather than a recurrence of the primary CRC (8). Despite guidelines recommending postoperative colonoscopy for CRC, the incidence of mCRC has not decreased over the past decade (5–7), with the cumulative incidence ranging from 1% to 4% (7). The etiology of mCRC remains undetermined, but missed lesions resulting from incomplete colonoscopy are a major contributing factor (6, 8, 9). For CRC patients who undergo incomplete preoperative colonoscopy, a prompt re-examination via colonoscopy following surgery can facilitate the timely detection and removal of polyps, thereby preventing the occurrence of mCRC.

The aim of this study was to explore the risk factors associated with CRC complicated by synchronous premalignant polyps, particularly high-risk polyps.

## Patients and methods

### Study population

This retrospective case-control study involved CRC patients who were treated at the Six Affiliated Hospital, Sun Yat-sen University (Guangzhou, China) from December 2014 to September 2018. Approval was obtained from the ethics committee of the Sixth Affiliated Hospital, Sun Yat-sen University. The procedures used in this study adhere to the tenets of the Declaration of Helsinki. Patients with CRC were identified through inpatient and outpatient discharge diagnoses, colonoscopy reports, or pathology reports from the hospital. All eligible patients had undergone at least one colonoscopy at the Six Affiliated Hospital, Sun Yat-sen University, which included an initial colonoscopy either preoperatively or within six months after surgery. Patients with Peutz-Jeghers syndrome (PJS) or familial adenomatous polyposis (FAP) were excluded from the data collection process.

### Case and control identification

We collected the clinical records, colonoscopy reports, and pathology reports of 6,674 CRC patients from the Six Affiliated Hospital, Sun Yat-sen University. Potential participants were excluded for the following reasons: no recorded preoperative or postoperative colonoscopy within six months (*n* = 1,979), FAP (*n* = 35), or PJS (*n* = 1). Ultimately, a total of 4,659 eligible patients were included in the study. Based on the endoscopy and pathology reports, the study participants were classified into three groups. Participants with at least one synchronous polyp measuring ≥10 mm or exhibiting a villous/tubulovillous component or high-grade dysplasia were categorized as high-risk polyp cases. Participants with synchronous polyps that did not meet the criteria for high-risk polyps were classified as low-risk polyp cases. Controls were participants who had no synchronous polyps detected during the colonoscopy.

### Definition of variables

We documented patient, tumor, and polyp information on a standardized form utilizing pre-defined definitions of variables: (1) Patient information encompassed admission number (AD), gender, age, admission date, and body mass index (BMI) (Chinese standard). (2) Tumor characteristics comprised anatomical location, pathologic stage, DNA mismatch repair (MMR), PIK3CA, NRAS, BRAF, and KRAS gene mutation status. (3) Polyp characteristics included number, size, pathological diagnosis results, and anatomical location.

Age was categorized as <50 and ≥50 years old. BMI was categorized as <18.5, 18.5–23.9, and >23.9 kg/m^2^ in accordance with Chinese classification standards. Tumor anatomical locations were categorized as colon or rectum. Pathologic stage was determined based on the American Joint Committee on Cancer (AJCC) TNM Staging Classification for Carcinoma of the Colon and Rectum (Eighth Edition) (10). In our statistical analysis, patients with stage I and II were consolidated as early-stage patients, while those with stage III and IV were classified as advanced patients. MMR was categorized as deficient MMR (dMMR) and proficient MMR (pMMR). Tumor gene mutation status was classified as mutant or wild-type.

### Statistical analysis

The demographic and disease characteristics of the patients were described using descriptive statistics. Proportions were used to present all dichotomous variables. The association between categorical variables based on baseline characteristics was tested using Pearson's *χ*^2^ test. Univariate analysis and multivariate logistic regression were utilized to calculate odds ratios (ORs) and corresponding 95% confidence intervals (CIs) for all potential risk factors. A significance level of *P *< 0.05 was considered statistically significant for all analyses, and two-sided tests were conducted. All statistical analyses were performed using SPSS software version 25.0 for Windows.

## Results

**Figure 1:**
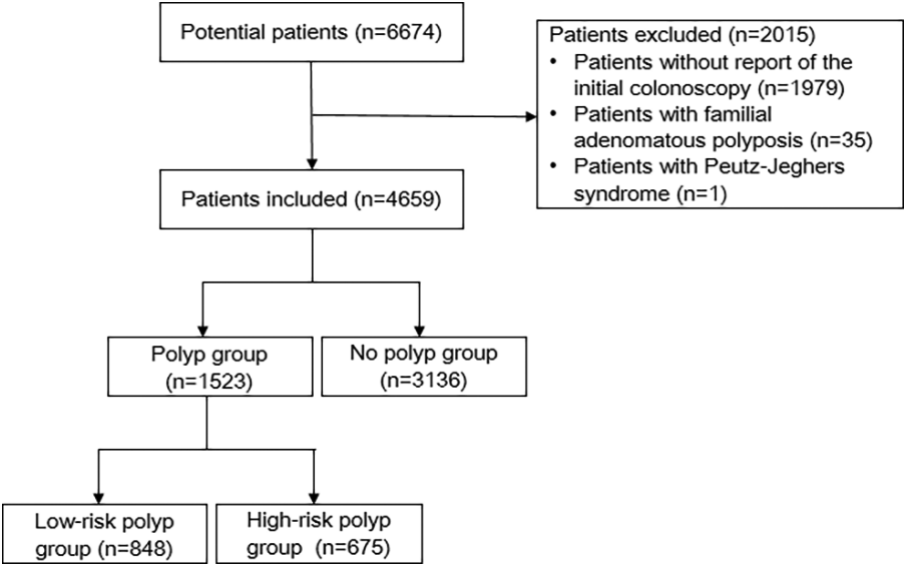
Flow diagram of the study.

A total of 6,674 CRC patients were initially considered as potential participants for our study. The flow diagram outlining the study process is presented in Figure 1. Ultimately, based on the inclusion and exclusion criteria, a total of 4,659 patients were included in our analysis. Table 1 illustrates the characteristics of CRC patients with or without synchronous polyps. 1,523 patients had polyps, among which 1,295 were found with polyp in preoperative colonoscopy. The median number of polyp found was 2. 675 had high-risk polyps, while 848 had low-risk polyps. Patients with polyps were found to be significantly older, predominantly male, and had higher BMI compared to those without polyps. Notably, a higher proportion of patients with rectal tumors were observed in the no polyp group. Additionally, the polyp group had a higher proportion of patients with early-stage tumors. Furthermore, BRAF mutant tumors were more prevalent in the no polyp group. No significant differences were observed in terms of MMR, KRAS mutation status, NRAS mutation status, and PIK3CA mutation status.

**Table 1:** Characteristics of colorectal cancer patients with or without synchronous polyp.

Variables	Total (*n* = 4,659)				*P* value^a^
	No polyp group (*n* = 3,136) (*n*, %)	Polyp group (*n* = 1,523) (*n*, %)	Low-risk polyp group (*n* = 848) (*n*, %)	High-risk polyp group (*n* = 675) (*n*, %)	
Sex					<0.001
Female	1,306 (41.6)	414 (27.2)	241 (28.4)	173 (25.6)	
Male	1,830 (58.4)	1,109 (72.8)	607 (71.6)	502 (74.4)	
Age at CRC diagnosis (years)					<0.001
<50	854 (27.2)	205 (13.5)	127 (15.0)	78 (11.6)	
≥50	2,282 (72.8)	1,318 (86.5)	721 (85.0)	597 (88.4)	
BMI (kg/m^2^)					<0.001
<18.5	318 (10.1)	115 (7.6)	64 (7.5)	51 (7.6)	
18.5–23.9	1,839 (58.6)	819 (53.8)	434 (51.2)	385 (57.0)	
>23.9	973 (31.0)	585 (38.4)	348 (41.0)	237 (35.1)	
NA	6 (0.2)	4 (0.3)	2 (0.2)	2 (0.3)	
Tumor location					<0.001
Rectum	1,733 (55.3)	738 (48.5)	436 (51.4)	302 (44.7)	
Colon	1,350 (43.0)	739 (48.5)	399 (47.1)	340 (50.4)	
Multiple primary carcinoma	53 (1.7)	46 (3.0)	13 (1.5)	33 (4.9)	
Pathologic stage					<0.001
Ⅰ+Ⅱ	1,585 (50.5)	894 (58.7)	490 (57.8)	404 (59.9)	
III	974 (31.1)	398 (26.1)	217 (25.6)	181 (26.8)	
IV	464 (14.8)	184 (12.1)	117 (13.8)	67 (9.9)	
NA	113 (3.6)	47 (3.1)	24 (2.8)	23 (3.4)	
Histological style					0.220
Adenocarcinoma	2,888 (92.1)	1,418 (93.1)			
Others^b^	248 (7.9)	105 (6.9)			
DNA mismatch repair (MMR)					0.346
pMMR	2,484 (79.2)	1,229 (80.7)	688 (81.1)	541 (80.1)	
dMMR	308 (9.8)	130 (8.5)	68 (8.0)	62 (9.2)	
NA	344 (11.0)	164 (10.8)	92 (10.8)	72 (10.7)	
PIK3CA					0.110
Wild-type	1,834 (58.5)	939 (61.7)	512 (60.4)	427 (63.3)	
Mutant	255 (8.1)	110 (7.2)	62 (7.3)	48 (7.1)	
NA	1,047 (33.4)	474 (31.1)	274 (32.3)	200 (29.6)	
NRAS					0.112
Wild-type	2,007 (64.4)	1,006 (66.1)	557 (65.7)	449 (66.5)	
Mutant	66 (2.1)	41 (2.7)	17 (1.0)	24 (3.6)	
NA	1,063 (33.9)	476 (31.3)	274 (32.3)	202 (29.9)	
BRAF					0.028
Wild-type	2,023 (64.5)	1,028 (67.5)	561 (66.2)	467 (69.2)	
Mutant	67 (2.1)	19 (1.2)	13 (1.5)	6 (0.9)	
NA	1,046 (33.4)	476 (31.3)	274 (32.3)	202 (29.9)	
KRAS					0.207
Wild-type	1,138 (36.3)	586 (38.5)	331 (39.0)	255 (37.8)	
Mutant	953 (30.4)	466 (30.6)	245 (28.9)	221 (32.7)	
NA	1,045 (33.3)	471 (30.9)	272 (32.1)	199 (29.5)	

^a^ In the Person's *χ*^2^ test, the significance was based on the differences between the no polyp group and polyp group.

^b^ Others include mucinous adenocarcinoma, signet-ring cell carcinoma and undifferentiated carcinoma.

CRC, colorectal cancer; BMI, body mass index; NA, not available; pMMR, proficient MMR; dMMR, deficient MMR.

### Risk factors for synchronous high-risk polyps

Univariate analysis and multivariate logistic regression analyses were conducted to identify the risk factors associated with synchronous high-risk polyps, as presented in Table 2. In the univariate analysis, male sex, age ≥50, colon tumors, early-stage tumors, NRAS mutant tumors, and BRAF wild-type tumors were significantly associated with an increased risk of high-risk polyps. However, BMI, MMR, PIK3CA mutation status, and KRAS mutation status showed no association with high-risk polyps. All variables that showed statistical significance in the univariate analysis (*P *< 0.05) were included in the multivariate logistic regression analysis. After adjusting for confounding factors, male sex (OR, 2.07; 95% CI, 1.71–2.50), age ≥50 (OR, 2.77; 95% CI, 2.15–3.56), colon tumors (OR, 1.53; 95% CI, 1.28–1.83), early-stage tumors (OR, 1.46; 95% CI, 1.23–1.75), NRAS mutant tumors (OR, 1.66; 95% CI, 1.01–2.72), and BRAF wild-type tumors (OR, 2.43; 95% CI, 1.03–5.70) remained significantly associated with a higher risk of high-risk polyps. Consequently, we identified male sex, age ≥50, colon tumors, early-stage tumors, NRAS mutant tumors, and BRAF wild-type tumors as independent risk factors for synchronous high-risk polyps.

**Table 2:** Univariate and multivariate logistic regression analysis on risk factors for colorectal cancer complicated with high-risk polyps.

Variables		Univariate			Multivariate		
	High-risk polyps, *n* (%)	OR	95% CI	*P* value	OR	95% CI	*P* value
Sex							
Female	173 (25.6)	1 (ref)			1 (ref)		
Male	502 (74.4)	2.07	1.72–2.50	<0.001	2.07	1.71–2.50	<0.001
Age at CRC diagnosis (years)							
<50	78 (11.6)	1 (ref)			1 (ref)		
≥50	597 (88.4)	2.86	2.23–3.67	<0.001	2.77	2.15–3.56	<0.001
BMI (kg/m^2^)							
<18.5	51 (7.6)	0.77	0.56–1.05	0.098			
18.5–23.9	385 (57.0)	1 (ref)					
>23.9	237 (35.1)	1.16	0.97–1.39	0.098			
Tumor location							
Rectum	302 (44.7)	1 (ref)			1 (ref)		
Colon	340 (50.4)	1.45	1.22–1.71	<0.001	1.53	1.28–1.83	<0.001
Multiple primary carcinoma	33 (4.9)	3.57	2.28–5.61	<0.001	3.32	2.09–5.30	<0.001
Pathologic stage							
Ⅰ+Ⅱ	404 (59.9)	1.48	1.24–1.76	<0.001	1.46	1.23–1.75	<0.001
Ⅲ+Ⅳ	248 (36.7)	1 (ref)			1 (ref)		
DNA mismatch repair (MMR)							
pMMR	541 (80.1)	1 (ref)					
dMMR	62 (9.2)	0.92	0.69–1.23	0.592			
PIK3CA							
Wild-type	427 (63.3)	1 (ref)					
Mutant	48 (7.1)	0.81	0.58–1.12	0.201			
NRAS							
Wild-type	449 (66.5)	1 (ref)			1 (ref)		
Mutant	24 (3.6)	1.63	1.01–2.62	0.047	1.66	1.01–2.72	0.046
BRAF							
Wild-type	467 (69.2)	2.58	1.11–5.98	0.027	2.43	1.03–5.70	0.042
Mutant	6 (0.9)	1 (ref)			1 (ref)		
KRAS							
Wild-type	255 (37.8)	1 (ref)					
Mutant	221 (32.7)	1.04	0.85–1.26	0.736			

CRC, colorectal Cancer; BMI, body mass index; OR, odds ratio; 95% CI, 95 percent confidence interval; ref, reference; pMMR, proficient MMR; dMMR, deficient MMR.

### Risk factors for synchronous low-risk polyps

We employed the same methodology to analyze the risk factors associated with synchronous low-risk polyps, and the results are presented in Table 3. In the univariate analysis, male sex, age ≥ 50, BMI > 23.9, colon tumors, and early-stage tumors were significantly associated with an increased risk of low-risk polyps. However, MMR, KRAS mutation status, NRAS mutation status, BRAF mutation status, and PIK3CA mutation status showed no association with low-risk polyps. After adjusting for confounding variables using multivariate logistic regression analysis, male sex (OR, 1.79; 95% CI, 1.52–2.12), age ≥ 50 (OR, 2.07; 95% CI, 1.69–2.55), BMI > 23.9 (OR, 1.52; 95% CI, 1.29–1.79), colon tumors (OR, 1.21; 95% CI, 1.03–1.41), and early-stage tumors (OR, 1.31; 95% CI, 1.12–1.54) remained significantly associated with a higher risk for synchronous low-risk polyps.

**Table 3:** Univariate and multivariate logistic regression analysis on risk factors for colorectal cancer complicated with low-risk polyps.

Variables		Univariate			Multivariate		
	Low-risk polyps, *n* (%)	OR	95% CI	*P* value	OR	95% CI	*P* value
Sex							
Female	241 (28.4)	1 (ref)			1 (ref)		
Male	607 (71.6)	1.80	1.52–2.12	<0.001	1.79	1.52–2.12	<0.001
Age at CRC diagnosis (years)							
<50	127 (15.0)	1 (ref)			1 (ref)		
≥50	721 (85.0)	2.13	1.73–2.61	<0.001	2.07	1.69–2.55	<0.001
BMI (kg/m^2^)							
<18.5	64 (7.5)	0.85	0.64–1.14	0.279	0.93	0.69–1.24	0.622
18.5–23.9	434 (51.2)	1 (ref)			1 (ref)		
>23.9	348 (41.0)	1.52	1.29–1.78	<0.001	1.52	1.29–1.79	<0.001
Tumor location							
Rectum	436 (51.4)	1 (ref)			1(ref)		
Colon	399 (47.1)	1.18	1.01–1.37	0.039	1.21	1.03–1.41	0.020
Multiple primary carcinoma	13 (1.5)	0.98	0.53–1.80	0.936	0.95	0.51–1.77	0.863
Pathologic stage							
Ⅰ+Ⅱ	490 (57.8)	1.33	1.14–1.56	<0.001	1.31	1.12–1.54	0.001
Ⅲ+Ⅳ	334 (39.4)	1 (ref)			1 (ref)		
DNA mismatch repair (MMR)							
pMMR	688 (81.1)	1 (ref)					
dMMR	68 (8.0)	0.80	0.61–1.05	0.107			
PIK3CA							
Wild-type	512 (60.4)	1 (ref)					
Mutant	62 (7.3)	0.87	0.65–1.17	0.357			
NRAS							
Wild-type	557 (65.7)	1 (ref)					
Mutant	66 (2.1)	0.93	0.54–1.60	0.787			
BRAF							
Wild-type	561 (66.2)	1 (ref)					
Mutant	13 (1.5)	1.43	0.78–2.61	0.244			
KRAS							
Wild-type	331 (39.0)	1 (ref)					
Mutant	245 (28.9)	0.88	0.73–1.07	0.194			

CRC, colorectal Cancer; BMI, body mass index; OR, odds ratio; 95%CI, 95 percent confidence interval; ref, reference; pMMR, proficient MMR; dMMR, deficient MMR.

## Discussion

Preoperatively missed synchronous premalignant polyps are a significant contributor to the development of mCRC in CRC patients. To identify potential strategies for preventing mCRC, we investigated several risk factors associated with CRC complicated by premalignant polyps. In a multivariate analysis, we identified male sex, age ≥50, colon tumors, early-stage tumors, NRAS mutant tumors, and BRAF wild-type tumors as risk factors for CRC patients with high-risk polyps. Additionally, we analyzed the risk factors of CRC complicated by low-risk polyps and found that the risk was increased in patients with male sex, age ≥ 50, BMI > 23.9, colon tumors, and early-stage tumors.

Following surgery and adjuvant treatment, such as radiation therapy and chemotherapy, many patients with non-metastatic CRC are cured. However, the morbidity and mortality rates among CRC patients have not declined in recent years, potentially due to the occurrence of mCRC. Although many studies have attempted to identify the causes of mCRC, few have been reported. Research has shown that part of the metachronous cancers may be caused by missed lesions (8). Some studies have reported that the miss rate of premalignant polyps found during colonoscopy ranges from 12% to 47% (11–15). Therefore, our study aimed to identify the risk factors associated with CRC complicated by premalignant polyps to identify patients who are more likely to have missed lesions.

Our study confirmed that male and advanced age are risk factors for CRC complicated with synchronous high-risk polyps, which is consistent with the findings of Kazushige Kawai et al. (16). It is well-known that men have a higher incidence of colorectal cancer compared to women. Additionally, the average age of male patients is higher than that of female patients, and advanced age has been established as a strong risk factor for mCRC (17–19). However, current postoperative surveillance practices for CRC patients do not take gender or age into consideration (4–6, 9). Considering our findings, it may be worth considering whether older men should undergo more frequent postoperative surveillance. This could potentially help in reducing the development of mCRC.

In our study, we found that patients with early-stage tumors had a higher risk of synchronous polyps than those with advanced tumors. Interestingly, patients with advanced tumors had a higher likelihood of inadequate colonoscopy due to bowel obstruction, which may result in an underestimation of the number of polyps present in these patients. Our results also showed that tumors located in the colon had a greater risk of synchronous polyps compared to tumors located in the rectum. Previous research has demonstrated that colon tumors are more frequently associated with microsatellite instability, overexpression of epiregulin, chromosomal instability, and epidermal growth factor receptor amplification compared to rectal tumors (20–24). These pathological features may contribute to the increased risk of synchronous polyps in patients with colon tumors.

Currently, several biomarkers play a crucial role in guiding clinicians towards making optimal treatment decisions for CRC patients. These biomarkers include PIK3CA, NRAS, BRAF, KRAS, and DNA mismatch repair genes. They help in the selection of individualized treatment plans based on their respective functions. In our study, we observed that NRAS mutant tumors and BRAF wild-type tumors were identified as risk factors for synchronous high-risk polyps in CRC patients. Traditionally, these biomarkers have been primarily used to guide the treatment of CRC patients. However, our findings indicate an additional role for them. By assessing the mutation status of NRAS and BRAF, we can potentially predict patients at higher risk of synchronous polyps and subsequently intensify postoperative surveillance for these individuals.

The prevention of postoperative CRC through the detection and removal of high-risk polyps is considered a crucial benefit of postoperative colonoscopy. Therefore, various expert guidelines recommend colonoscopy after radical surgery for CRC (4–6, 9). However, several studies have shown that intensive postoperative surveillance, including annual colonoscopies, does not provide a survival benefit compared to standard follow-ups (25–27). Additionally, colonoscopy is an invasive procedure that carries risks, including severe complications such as bleeding and perforation, which can even lead to death. Thus, it is essential to strike a balance between the risk of mCRC and complications when determining the optimal surveillance strategy after curative surgery, in order to avoid unnecessary colonoscopies. However, data on the risk factors for mCRC are limited and often conflicting, and current guidelines do not recommend risk stratification for postoperative endoscopic surveillance (9). In our study, we aimed to identify risk factors through multivariate analysis and stratify patients based on their risk of developing mCRC after surgery. The current findings suggest that male gender, age ≥50, colon tumors, early-stage tumors, NRAS mutant tumors, and BRAF wild-type tumors are risk factors for CRC with high-risk polyps. We recommend that CRC patients with these risk factors, especially those with multiple risk factors, undergo their first postoperative colonoscopy 6 months after curative surgery. Patients who do not have these risk factors can undergo postoperative colonoscopy within one year after surgery, following the standard follow-up protocol (25–27). This approach ensures that patients receive appropriate surveillance while avoiding unnecessary invasive procedures. A well-planned surveillance strategy can optimize the utilization of endoscopy resources, reduce the burden on the medical and health service systems, and promote efficiency. It is important to note that a high-quality index colonoscopy is crucial. If the bowel preparation is inadequate, the patient should undergo a repeat colonoscopy as soon as possible to detect any missed lesions. In cases where the preoperative colonoscopy was incomplete due to intestinal obstruction, a postoperative colonoscopy should also be performed within 6 months after surgery.

Our study possessed several strengths. Firstly, a key advantage was the substantial size of our study population, encompassing nearly 4,659 CRC patients. Secondly, we conducted a comprehensive review of the colonoscopy reports and pathology reports of each patient, categorizing them into the no polyp group, high-risk polyp group, and low-risk polyp group. This stratification was essential due to the differing risk of developing CRC between high-risk and low-risk polyps, with high-risk polyps serving as better predictors of mCRC occurrence. Thirdly, the variables we analyzed, such as sex, age, tumor location, and biomarkers, were readily available, rendering our research results more applicable in clinical practice. Several limitations need to be noted. One significant limitation of this study was its retrospective nature, meaning that we relied on data as recorded in clinical records, colonoscopy reports, and pathology reports. Colonoscopists' subjective estimation of polyp size may have introduced errors, potentially leading to an inflated number of high-risk polyp cases. Secondly, due to the retrospective design, incomplete clinical data were unavoidable. Thirdly, the exclusion of patients without colonoscopy reports may have resulted in selection bias.

## Conclusion

Male gender, advanced age, colon tumors, early-stage tumors, NRAS mutant tumors, and BRAF wild-type tumors were identified as characteristics associated with CRC complicated by high-risk polyps. Early follow-up of colonoscopy in patients exhibiting these characteristics is crucial to detect synchronous polyps early.

## Data Availability

The raw data supporting the conclusions of this article will be made available by the authors, without undue reservation.
